# Different phenotype of left atrial function impairment in patients with hypertrophic cardiomyopathy and hypertension: comparison of healthy controls

**DOI:** 10.3389/fcvm.2023.1027665

**Published:** 2023-05-10

**Authors:** Hongwen Li, Haibao Wang, Tingting Wang, Chaolong Jin, Minjie Lu, Bin Liu

**Affiliations:** ^1^ Department of Radiology, The First Affiliated Hospital of Anhui Medical University, Hefei, China; ^2^ Cardiac Imaging Center, The First Affiliated Hospital of Anhui Medical University, Hefei, China; ^3^ Department of Magnetic Resonance Imaging, Fuwai Hospital, National Center for Cardiovascular Diseases, Chinese Academy of Medical Sciences and Peking Union Medical College, Beijing, China; ^4^ Key Laboratory of Cardiovascular Imaging (Cultivation), Chinese Academy of Medical Sciences, Beijing, China

**Keywords:** atrial function, feature tracking, hypertrophic cardiomyopathy, hypertension, magnetic resonance imaging

## Abstract

**Background:**

The impairment of atrial function and atrial-ventricular coupling in diseases with left ventricular (LV) hypertrophy has been increasingly recognized. This study compares left atrium (LA) and right atrium (RA) function, as well as LA-LV coupling, in patients with hypertrophic cardiomyopathy (HCM) and hypertension (HTN) with preserved LV ejection fraction (EF), using cardiovascular magnetic resonance feature tracking (CMR-FT).

**Methods:**

Fifty-eight HCM patients, 44 HTN patients, and 25 healthy controls were retrospectively enrolled. LA and RA functions were compared among the three groups. LA-LV correlations were evaluated in the HCM and HTN groups.

**Results:**

LA reservoir (LA total EF, ɛs, and SRs), conduit (LA passive EF, ɛe, SRe), and booster pump (LA booster EF, ɛa, SRa) functions were significantly impaired in HCM and HTN patients compared to healthy controls (HCM vs. HTN vs. healthy controls: ɛs, 24.8 ± 9.8% vs. 31.3 ± 9.3% vs. 25.2 ± 7.2%; ɛe, 11.7 ± 6.7% vs. 16.8 ± 6.9% vs. 25.5 ± 7.5%; ɛa, 13.1 ± 5.8% vs. 14.6 ± 5.5% vs. 16.5 ± 4.5%, *p* < 0.05). Reservoir and conduit functions were more impaired in HCM patients compared to HTN patients (*p* < 0.05). LA strains demonstrated significant correlations with LV EF, LV mass index, LV MWT, global longitudinal strain parameters, and native T1 in HCM patients (*p* < 0.05). The only correlations in HTN were observed between LA reservoir strain (ɛs) and booster pump strain (ɛa) with LV GLS (*p* < 0.05). RA reservoir function (RA ɛs, SRs) and conduit function (RA ɛe, SRe) were significantly impaired in HCM and HTN patients (*p* < 0.05), while RA booster pump function (RA ɛa, SRa) was preserved.

**Conclusions:**

LA functions were impaired in HCM and HTN patients with preserved LV EF, with reservoir and conduit functions more affected in HCM patients. Moreover, different LA-LV couplings were apparent in two different diseases, and abnormal LA-LV coupling was emphasized in HTN. Decreased RA reservoir and conduit strains were evident in both HCM and HTN, while booster pump strain was preserved.

## 1. Introduction

Hypertrophic cardiomyopathy (HCM) and hypertension (HTN) are two common cardiovascular diseases involving left ventricular hypertrophy (LVH), characterized by distinct histological changes of replacement fibrosis and diffuse interstitial fibrosis (1, 2). Myocardial hypertrophy and diastolic dysfunction of the left ventricle (LV) are the main manifestations of these diseases (2, 3). Hemodynamically, the left atrium (LA) plays a crucial role in modulating LV diastolic filling through the following basic functional elements: (1) reservoir function (collection of pulmonary venous return during ventricular systole); (2) conduit function (passage of blood to the LV during early diastole); and (3) contractile booster pump function (augmentation of ventricular filling during late diastole). LA remodeling and dysfunction have been increasingly recognized and are closely related to atrial fibrillation and the development of cardiovascular disease, as demonstrated by conventional indices such as LA size and volume (4–7) and the latest strain measurements (8, 9). Deformation studies have shown that LA functions, particularly reservoir and conduit functions, are impaired prior to LA enlargement in the early stages of HCM and HTN (8–12). However, most of these findings have been reported in separate studies and have rarely been compared together.

Although both HCM and HTN present with pathological LVH, they are two distinct diseases with specific pathophysiological mechanisms. HCM is the most common inheritable heart disorder activated via genetic pathways (13, 14), while HTN is an acquired chronic disease initiated by pressure-related LV diastolic dysfunction triggered by increased afterload (12, 15). Further exploration of LA-LV coupling in these two different diseases has gradually gained attention but has not been sufficiently investigated thus far. We hypothesize that impaired LA-LV coupling plays a key role in LA dysfunction.

On the other hand, the right heart, previously considered a “dispensable” part of the heart, has recently become a research hotspot in pathophysiological conditions such as heart failure and pulmonary hypertension (16). Numerous studies have revealed that right ventricular (RV) hypertrophy and diastolic dysfunction are essential components of cardiac damage in HCM and systemic hypertension, possibly secondary to ventricular interaction (2, 17). However, data focusing on right atrial (RA) function in diseases with LVH are limited, even though RA plays a critical role in modulating RV diastolic filling (18). It has been reported that RA deformation is significantly impaired in hypertensive patients who are untreated or ineffectively treated using echocardiographic speckle tracking (STE) (19). CMR-feature tracking (CMR-FT) is a novel offline technique for myocardial deformation evaluation based on routinely acquired balanced steady-state free precession sequence (SSFP) cine images. It offers a larger field of view encompassing the four chambers and has superior reproducibility compared to STE (20, 21). Recent studies have demonstrated that CMR-FT can be used for RA strain in various diseases (22–24).

Therefore, the present study aims to compare LA function between patients with HCM and HTN with preserved EF and further explores LA-LV coupling in these two diseases. We also investigate the feasibility of CMR-FT in RA deformation assessment and whether early RA dysfunction can be detected in HCM and HTN patients.

## 2. Materials and methods

### 2.1. Patient population

We retrospectively enrolled 58 consecutive HCM and 44 HTN patients between January 2019 and November 2022. The HCM inclusion criteria were as follows: CMR demonstrating LVH (maximal wall thickness ≥15 mm in adults or ≥13 mm in adults with relatives who had HCM) without other hypertrophy-causing diseases (25). HTN was defined as a SBP >140 mmHg and/or a DBP >90 mmHg based on at least two office readings (26). HTN patients included individuals receiving antihypertensive treatment and newly diagnosed patients. SBP and DBP measurements were obtained during hospitalization or outpatient visits. The exclusion criteria were: (1) claustrophobia, impaired renal function, pacemaker/defibrillator devices, or other metallic implants; (2) LV EF <50%; (3) atrial fibrillation; (4) history of septal myectomy or alcoholic septal ablation; (5) coronary artery stenosis >50% confirmed by coronary CT angiography or coronary angiography; (6) severe valvular diseases. Twenty-five normotensive subjects (11 females, 14 males) with no history of cardiovascular disease and normal physical examination results were selected as the control group. The local institutional ethics committee approved this study, and all subjects provided written informed consent.

### 2.2. CMR protocol

#### 2.2.1. Image acquisition

All CMR images were obtained using two clinical 3.0-T MR scanners (Magnetom Prisma, Siemens, Erlangen, Germany and Ingenia, Philips Healthcare, Best, the Netherlands). Transverse dark blood images were acquired with the following parameters: slice thickness: 8 mm; TR: 882 ms; TE: 40 ms; FOV: 344 mm × 343 mm. Balanced SSFP breath-held cine images were acquired in the two-chamber, three-chamber, four-chamber, and 8–11 equidistant short-axis planes, covering the entire LV and RV. Typical imaging parameters included: slice thickness: 8 mm; TR: 3.4 ms; TE: 1.1–1.5 ms; FOV: 360 mm × 315 mm; spatial resolution: 1.3 mm × 1.3 mm × 8.0 mm; flip angle: 60–70°; temporal resolution 42 ms.

#### 2.2.2. Image analysis

Conventional size parameters were obtained from dark blood images and balanced SSFP cine images. LV EDD was measured at the papillary level of LV short-axis cine images, and RV EDD was measured on the extension cord of the left measuring line. MWT was defined as end-diastolic wall thickness selected in the thickest segment from the LV short-axis cines without involving trabeculations from both ventricles. AP diameters of LA and RA were measured on transverse dark blood images.

Functional and strain analyses were performed on balanced SSFP cine images using dedicated software (cvi42; Circle Cardiovascular Imaging Inc., Calgary, Canada, version 5.5). The following LV functional parameters were calculated from all of the phases on the short-axis and three long-axis cine images in end-systole and end-diastole: LV EDV, LV ESV, LV EF, and LV mass. The following RV functional parameters were calculated from all of the phases on the short-axis and four-chamber cine images in end-systole and end-diastole: RV EDV, RV ESV, RV EF, and RV mass. Papillary muscles were included in the volume and excluded from the mass calculations.

LV and RV strain measurements were carried out based on long-axis images by tracing endocardial and epicardial borders at end-systole and end-diastole. Endocardial and epicardial borders were semi-automatically detected and manually corrected, excluding the papillary muscles. Then, all frames throughout the entire cardiac cycle were propagated. LV GLS was derived from two-, three-, and four-chamber views, and RV GLS was derived from a four-chamber view. Associated peak global systolic and diastolic strain rates (SRs) were obtained simultaneously. Native T1 mapping of LV was obtained from the basal and mid-ventricular short-axis sections before contrast medium administration.

LA volumes were calculated using the biplane area–length method (27). Manual tracking of the LA outline and length was performed in two- and four-chamber views, excluding pulmonary veins and the LA appendage. RA volumes were calculated using the single-plane area–length method (28). Manual tracking of the RA area and length was performed in a four-chamber view (24). The LA volumes indexed based on BSA were assessed at LV end-systole (LAV max), at LV diastole before LA contraction (LAV pac), and at late LV diastole after LA contraction (LAV min). The RA volumes indexed based on BSA were assessed at RV end-systole (RAV max), at RV diastole before RA contraction (RAV pac), and at late RV diastole after RA contraction (RAV min). Bi-atrial total, passive, and booster EFs were defined according to the following equations: EF total = (*V*_max_ − *V*_min_) × 100%/*V*_max_; EF passive = (*V*_max_ − *V*_pac_) × 100%/*V*_max_; and EF booster = (*V*_pac_ − *V*_min_) × 100%/*V*_pac_.

**Figure 1:**
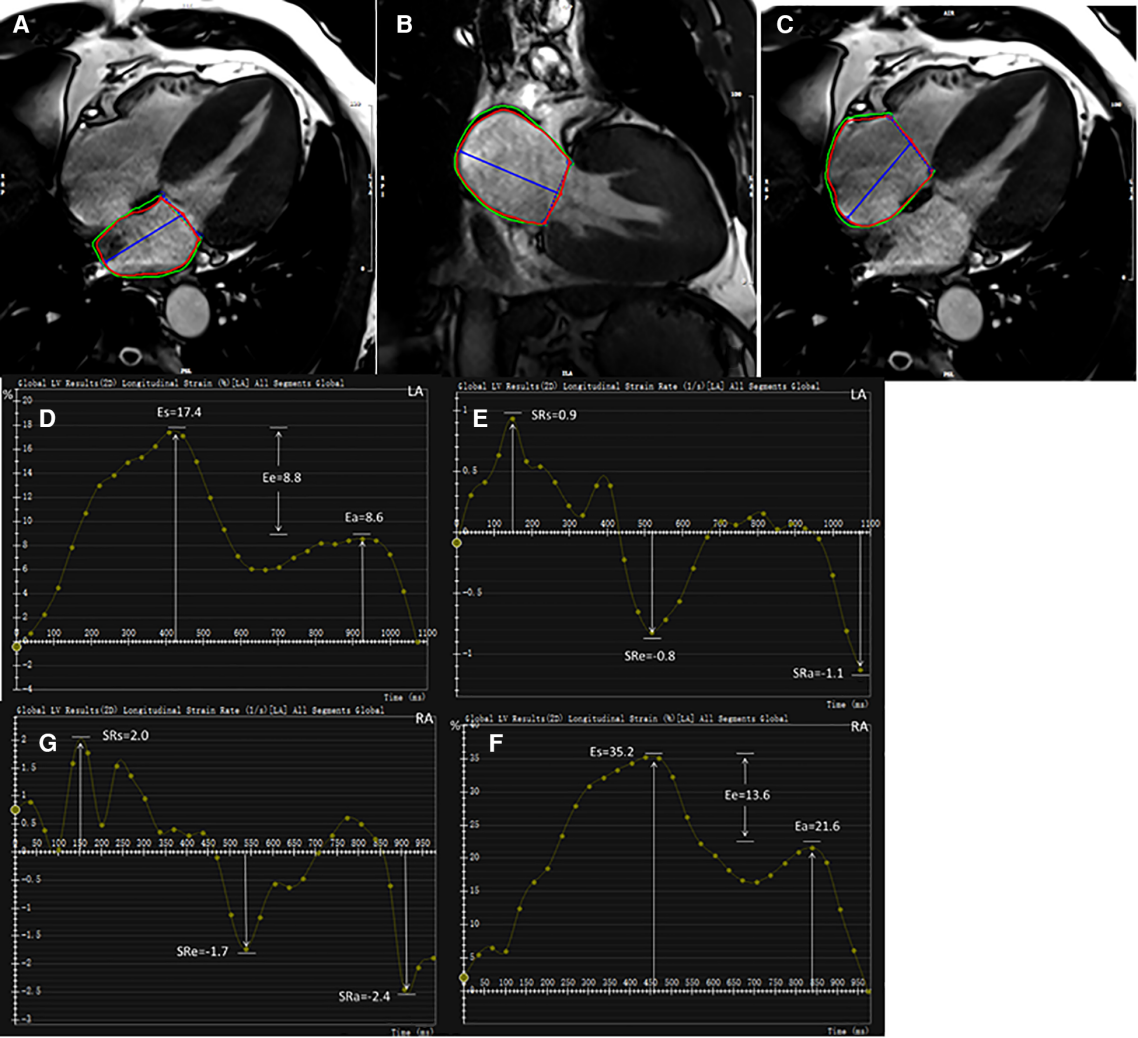
Representative HCM example for bi-atrial CMR feature tracking analysis. LA endocardial and epicardial contours at end-diastole shown on two- and four-chamber views (**A,B**). RA endocardial and epicardial borders at end-diastole shown on four-chamber view (**C**). Strain and strain rate parameters were obtained from strain curves (**D,F**) and strain rate curves (**E,G**), respectively. LA, left atrium; RA, right atrium; ɛs, total strain; ɛe, passive strain; ɛa, active strain; SRs, total strain rate; SRe, passive strain rate; SRa, active strain rate.

For strain analysis, LA endocardial and epicardial borders were tracked in two- and four-chamber views (Figures 1A,B). RA endocardial and epicardial borders were tracked in a four-chamber view (Figure 1C). The atrial borders were manually delineated in end-systole and end-diastole and then propagated to all frames automatically. Bi-atrial global longitudinal strain parameters were evaluated as ɛs (total strain, reflective of atrial reservoir function during ventricle systole), ɛe (passive strain, reflective of atrial conduit function during early ventricle diastole), and ɛa (active strain, reflective of atrial booster pump function during late ventricle diastole). Accordingly, their corresponding strain rate parameters were obtained as SRs (peak positive strain rate), SRe (peak early negative strain rate), and SRa (late peak negative strain rate). Five HCM and four HTN patients were excluded due to poor LA or RA tracking quality.

### 2.3. Reproducibility

Intra-observer reproducibility for global strain and SR parameters was assessed in 30 randomly selected subjects (10 HCM patients, 10 HTN patients, and 10 healthy controls) with a four-week interval between analyses (H.L. with four years of cardiovascular MRI experience). Inter-observer reproducibility was assessed in the same 30 subjects by comparing results from a second experienced observer (T.W. with five years of cardiovascular MRI experience).

### 2.4. Statistical analysis

Continuous variables were represented as means ± standard deviations. Categorical variables were represented as numbers and percentages. Comparisons of continuous variables among the three groups were performed using one-way ANOVA. Comparisons between the two groups (HCM vs. controls, HTN vs. controls, and HCM vs. HTN) were performed using independent *t*-test for normally distributed data or Mann–Whitney *U*-test for non-normally distributed data. Categorical variables were evaluated using chi-square test or Fisher's exact test. Pearson's or Spearman's correlation analysis was performed to investigate the relationship between LA strains and LV parameters. The correlation was considered weak if *r* was <0.5, moderate if *r* was between 0.5 and 0.7, and strong if *r* was >0.7 (29). Intra- and inter-observer variabilities of the atrial strain indices were evaluated using the Bland–Altman test. Reproducibility analysis was performed using intra-class correlation coefficients (ICCs) for absolute agreement and coefficients of variation (CoVs). All statistical analyses were performed with the IBM SPSS Statistical program for Windows (version 21.0, Armonk, NY), MedCalc software (version 15.0, Mariakerke, Belgium), and GraphPad Prism 8.0. *p*-Values of <0.05 were considered significant.

## 3. Results

### 3.1. Population characteristics

Table 1 lists the population characteristics of the study. BSA, BMI, and resting DBP were significantly higher in HTN patients than in HCM patients and healthy controls (all *p* < 0.05). No significant difference was observed in the rate of diabetes, resting SBP, and NYHA distribution between HCM and HTN patients.

**Table 1:** Study population characteristics.

	HCM (*n* = 58)	HTN (*n* = 44)	Healthy controls (*n* = 25)	*p* ^‡^
Age, years	52.1 ± 12.4*^,†^	47.7 ± 15.4	43.8 ± 15.5	**0** **.** **043**
Male gender, *n* (%)	40 (69.0%)	34 (77.3%)*	14 (56.0%)	0.183
BSA, m^2^	1.7 ± 0.2^†^	1.9 ± 0.2*	1.7 ± 0.2	**0**.**001**
BMI, kg/m^2^	25.0 ± 3.9^†^	27.3 ± 3.7*	23.4 ± 3.4	**<0**.**001**
Diabetes, *n* (%)	4 (6.9%)	5 (11.4%)	0 (0.0%)	0.494
Hypercholesterolemia, *n* (%)	14 (24.1%)	19 (43.2%)*	4 (16.0%)	**0**.**030**
Resting SBP, mmHg	137.7 ± 25.3	140.5 ± 23.4*	124.8 ± 13.1	0.128
Resting DBP, mmHg	80.5 ± 17.2^†^	88.8 ± 19.0*	75.7 ± 13.8	**0**.**032**
**NYHA**				
I, *n* (%)	46 (79.3%)	36 (81.8%)	–	
II, *n* (%)	10 (17.2%)	5 (11.4%)	–	
III, *n* (%)	2 (3.4%)	3 (6.8%)	–	
IV, *n* (%)	0 (0.0%)	0 (0.0%)	–	

Data are represented as means ± standard deviations or *n* (%). Bold values indicate statistical significance. HCM, hypertrophic cardiomyopathy; HTN, hypertension; BSA, body surface area; BMI, body mass index; SBP, systolic blood pressure; DBP, diastolic blood pressure. NYHA, New York Heart Association.

* Indicates *p* < 0.05 when compared to healthy controls.

^†^ Indicates *p* < 0.05 when compared to HTN patients.

^‡^ Significance of differences among three groups.

### 3.2. Conventional functional and strain parameters of LV and RV

HCM and HTN patients exhibited higher LV massi, LV EDD, LV MWT, RV massi, and RV EDD compared to healthy controls (all *p* < 0.05; Table 2). HCM patients had higher levels of LV EF, LV massi, LV MWT, RV EF, and RV EDD, as well as lower RV EDVi and RV ESVi compared to HTN patients and healthy controls (all *p* < 0.05).

**Table 2:** Conventional functional and strain parameters of left and right ventricles.

	HCM (*n* = 58)	HTN (*n* = 44)	Healthy controls (*n* = 25)	*p* ^‡^
**LV parameters**				
LVEF, %	67.3 ± 7.3*^,†^	63.2 ± 8.9	62.9 ± 6.2	**0**.**012**
LV massi, g/m^2^	102.7 ± 36.8*^,†^	75.1 ± 25.6*	49.7 ± 8.2	**<0**.**001**
LV EDD, mm	50.9 ± 5.6*	50.7 ± 5.9*	48.1 ± 3.1	0.063
LV MWT, mm	17.7 ± 4.0*^,†^	12.3 ± 2.5*	8.0 ± 1.3	**<0**.**001**
LV EDVi, ml/m^2^	80.1 ± 17.3	79.4 ± 17.1	78.5 ± 12.7	0.970
LV ESVi, ml/m^2^	26.2 ± 9.4	30.0 ± 13.2	29.3 ± 5.7	0.157
LV GLS, %	−10.3 ± 3.0*^,†^	−13.2 ± 2.7*	−17.9 ± 1.9	**<0**.**001**
LV sGLSR, s^−1^	−0.7 ± 0.2*^,†^	−0.8 ± 0.2	−1.0 ± 0.2	**<0**.**001**
LV dGLSR, s^−1^	0.6 ± 0.2*^,†^	0.7 ± 0.2*	0.9 ± 0.2	**<0**.**001**
Native T1, ms	1,300.1 ± 67.7*^,†^	1,253.7 ± 50.2	1,226.9 ± 18.8	**<0**.**001**
**RV parameters**				
RVEF, %	58.1 ± 9.0^†^	53.0 ± 11.6	56.9 ± 7.3	**0**.**033**
RV massi, g/m^2^	19.6 ± 4.5*	18.5 ± 4.6*	15.2 ± 4.6	**0**.**001**
RV EDD, mm	34.4 ± 4.5*^,†^	32.3 ± 4.8*	27.2 ± 5.3	**<0**.**001**
RV EDVi, ml/m^2^	59.8 ± 11.7*^,†^	67.9 ± 14.8	75.1 ± 9.9	**<0**.**001**
RV ESVi, ml/m^2^	24.9 ± 7.6*^,†^	32.2 ± 11.6	32.3 ± 6.6	**<0**.**001**
RV GLS, %	−16.7 ± 8.5*	−18.8 ± 4.5	−19.3 ± 2.9	0.138
RV sGLSR, s^−1^	−1.2 ± 0.7	−1.2 ± 0.3	−1.3 ± 0.3	0.696
RV dGLSR, s^−1^	1.1 ± 0.5	1.1 ± 0.3	1.2 ± 0.3	0.338

Data are represented as means ± standard deviations; Bold values indicate statistical significance.

LV, left ventricular; RV, right ventricular; EF, ejection fraction; massi, mass indexed by body surface area; EDD, end-diastolic diameter; MWT, maximal wall thickness; EDVi: end-diastolic volume indexed by body surface area; ESVi, end-systolic volume indexed by body surface area; GLS, global longitudinal strain; sGLSR, peak systolic global longitudinal strain; dGLSR, peak diastolic global longitudinal strain rate. ECV, extracellular volume.

* Indicates *p* < 0.05 when compared to healthy controls.

^†^ Indicates *p* < 0.05 when compared to HTN patients.

^‡^ Significance of differences among three groups.

Impaired LV GLS and peak diastolic global longitudinal strain rate (dGLSR) were observed in HCM and HTN patients compared to healthy controls. Furthermore, HCM patients had lower levels of GLS, sGLSR, and dGLSR compared to HTN patients (HCM vs. HTN vs. healthy controls: LV GLS, −10.3 ± 3.0% vs. −13.2 ± 2.7% vs. −17.9 ± 1.9%, all *p* < 0.05). Native T1 was elevated in HCM patients, but no significant difference was noted in HTN patients compared to healthy controls (HCM vs. HTN vs. healthy controls: 1,300.1 ± 67.7 ms vs. 1,253.7 ± 50.2 ms vs. 1,226.9 ± 18.8 ms, *p* < 0.05). Impaired RV GLS was observed in HCM patients, but no significant difference was found in HTN patients (HCM vs. HTN vs. healthy controls: −16.7 ± 8.5% vs. −18.8 ± 4.5% vs. −19.3 ± 2.9%, *p* > 0.05).

### 3.3. Volumetric and strain parameters of LA and RA

HCM and HTN patients had larger AP diameters of LA and RA compared to healthy controls, with HCM patients showing the highest values (*p* < 0.05). Table 3 displays the volumetric and strain parameters of LA and RA. HCM patients had the largest LA volumes, including *V*_maxi_, *V*_paci_, and *V*_mini_, followed by HTN patients and healthy controls (*p* < 0.05).

**Table 3:** Volumetric and strain parameters of left and right atria.

	HCM (*n* = 58)	HTN (*n* = 44)	Healthy Controls (*n* = 25)	*p* ^‡^
LA AP diameter, mm	39.3 ± 9.9*^,†^	34.3 ± 9.8*	30.3 ± 5.6	**<0** **.** **001**
RA AP diameter, mm	49.7 ± 5.7*^,†^	45.1 ± 6.1*	41.2 ± 5.6	**<0**.**001**
**LA volumetric parameters**				
LA *V*_maxi_, ml/m^2^	47.1 ± 16.7*^,†^	37.8 ± 12.6	33.7 ± 8.2	**<0**.**001**
LA *V*_paci_, ml/m^2^	40.4 ± 15.8*^,†^	30.4 ± 11.2*	25.2 ± 7.2	**<0**.**001**
LA *V*_mini_, ml/m^2^	25.3 ± 12.9*^,†^	17.1 ± 7.6*	12.3 ± 3.5	**<0**.**001**
**LA reservoir function**				
LA total EF, %	48.4 ± 11.6*^,†^	55.8 ± 8.4*	63.3 ± 7.2	**<0**.**001**
ɛs, %	24.8 ± 9.8*^,†^	31.3 ± 9.3*	41.9 ± 9.1	**<0**.**001**
SRs, s^−1^	1.3 ± 0.5*^,†^	1.6 ± 0.5*	1.9 ± 0.5	**<0**.**001**
**LA conduit function**				
LA passive EF, %	14.9 ± 8.2*^,†^	20.5 ± 8.7*	25.7 ± 6.3	**<0**.**001**
ɛe, %	11.7 ± 6.7*^,†^	16.8 ± 6.9*	25.5 ± 7.5	**<0**.**001**
SRe, s^−1^	−1.0 ± 0.5*^,†^	−1.6 ± 0.6*	−2.7 ± 0.9	**<0**.**001**
**LA booster pump function**				
LA booster EF, %	39.2 ± 13.6*	43.4 ± 13.9*	50.3 ± 9.8	**0**.**002**
ɛa, %	13.1 ± 5.8*	14.6 ± 5.5*	16.5 ± 4.5	**0**.**035**
SRa, s^−1^	−1.4 ± 0.6*	−1.6 ± 0.9*	−1.9 ± 0.5	**0**.**009**
**RA volumetric parameters**				
RA *V*_maxi_, ml/m^2^	33.6 ± 10.8	35.9 ± 9.8	37.1 ± 8.6	0.300
RA *V*_paci_, ml/m^2^	29.2 ± 8.9	28.6 ± 8.8	29.2 ± 7.8	0.932
RA *V*_mini_, ml/m^2^	16.9 ± 7.3	17.1 ± 6.7	18.7 ± 5.2	0.513
**RA reservoir function**				
RA total EF, %	51.3 ± 11.2	51.3 ± 9.5	50.8 ± 7.9	0.932
ɛs,%	31.8 ± 12.9*	35.1 ± 12.2*	41.3 ± 10.2	**0**.**006**
SRs, s^−1^	1.8 ± 0.7*	1.9 ± 0.7*	2.5 ± 0.8	**0**.**001**
**RA conduit function**				
RA passive EF, %	14.7 ± 5.9*^,†^	20.4 ± 6.7	23.0 ± 7.4	**<0**.**001**
ɛe, %	15.9 ± 8.2*^,†^	19.8 ± 8.5*	25.6 ± 9.0	**<0**.**001**
SRe, s^−1^	−1.3 ± 0.7*^,†^	−1.6 ± 0.6*	−2.1 ± 0.9	**<0**.**001**
**RA booster pump function**				
RA booster EF, %	40.6 ± 12.2	38.7 ± 11.3	36.0 ± 12.1	0.078
ɛa, %	15.9 ± 7.5	15.3 ± 8.2	15.8 ± 6.3	0.911
SRa, s^−1^	−1.9 ± 0.9	−1.9 ± 0.8	−1.8 ± 0.8	0.767

Data are represented as means ± standard deviations. Bold values indicate statistical significance.

LA, left atrial; RA, right atrial; AP, anterior-posterior; *V*_maxi_, maximal volume indexed by body surface area; *V*_paci_, pre-atrial contractile volume indexed by body surface area; *V*_mini_, minimal volume indexed by body surface area; EF, emptying fraction; ɛs, total strain; ɛe, passive strain; ɛa, active strain; SRs, total strain rate; SRe, passive strain rate; SRa, active strain rate.

* Indicates *p* < 0.05 when compared to healthy controls.

^†^ Indicates *p* < 0.05 when compared to HTN patients.

^‡^ Significance of differences among three groups.

**Figure 2:**
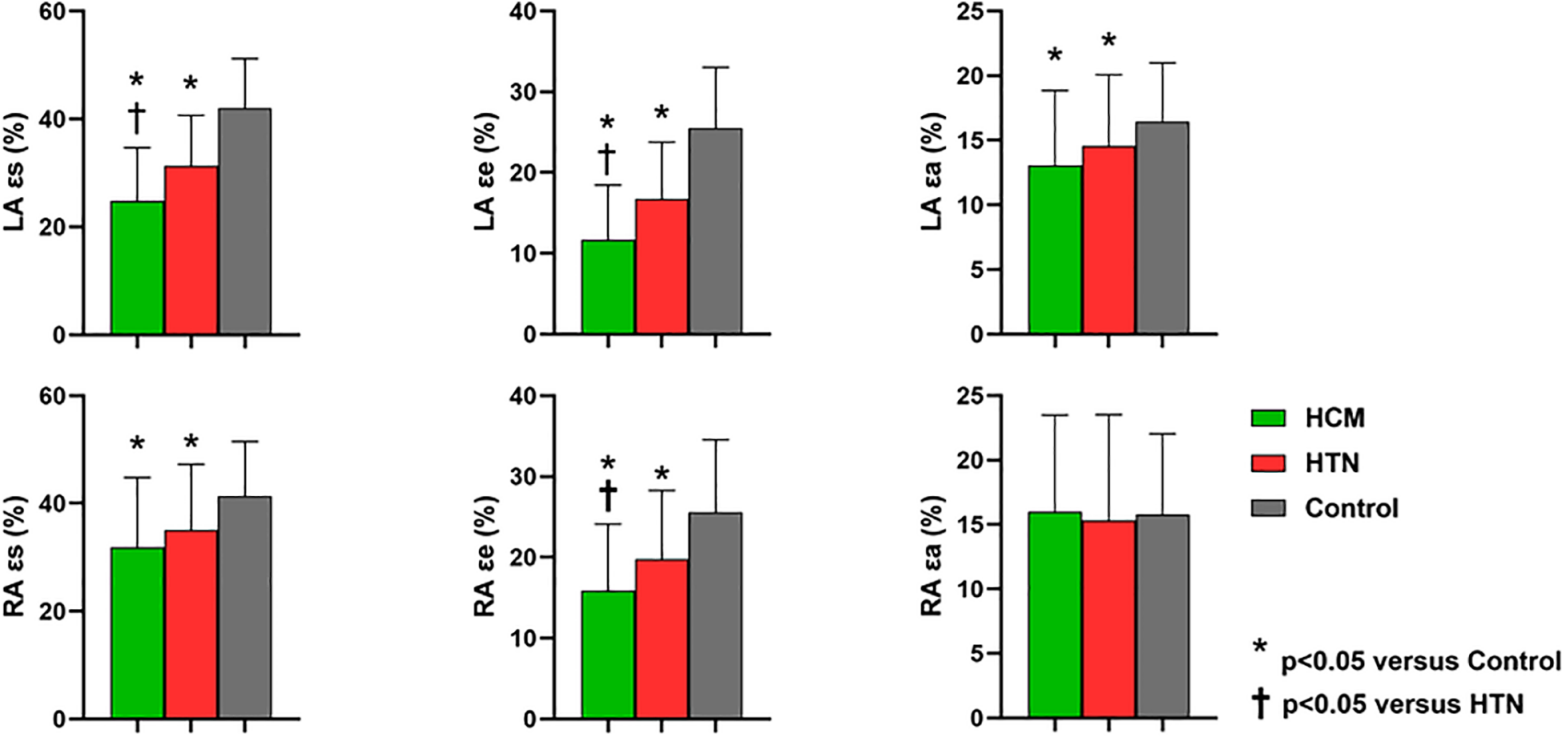
Comparisons of left and right atrial global strain parameters among HCM, HTN, and healthy control groups. LA, left atrial; RA, right atrial; ɛs, total strain; ɛe, passive strain; ɛa, active strain; SRs, total strain rate; SRe, passive strain rate; SRa, active strain rate. *Indicates *p* < 0.05 when compared to healthy controls; ^†^indicates *p* < 0.05 when compared to HTN patients.

LA reservoir function (LA total EF, ɛs, SRs), conduit function (LA passive EF, ɛe, SRe), and booster pump function (LA booster EF, ɛa, SRa) were significantly reduced in HCM and HTN patients compared to healthy controls (HCM vs. HTN vs. healthy controls: ɛs, 24.8 ± 9.8% vs. 31.3 ± 9.3% vs. 25.2 ± 7.2%; ɛe, 11.7 ± 6.7% vs. 16.8 ± 6.9% vs. 25.5 ± 7.5%; ɛa, 13.1 ± 5.8% vs. 14.6 ± 5.5% vs. 16.5 ± 4.5%, *p* < 0.05). Moreover, LA reservoir function (LA total EF, ɛs, SRs) and conduit function (LA passive EF, ɛe, SRe) were more impaired in HCM patients compared to HTN patients (*p* < 0.05; Figure 2).

Although RA volumetric parameters, including *V*_maxi_, *V*_paci_, and *V*_mini_, showed no significant differences among the three groups, RA reservoir strain (ɛs, SRs) and conduit strain (ɛe, SRe) were significantly impaired in HCM and HTN patients compared to healthy controls (HCM vs. HTN vs. healthy controls: ɛs, 31.8 ± 12.9% vs. 35.1 ± 12.2% vs. 41.3 ± 10.2%; ɛe, 15.9 ± 8.2% vs. 19.8 ± 8.5% vs. 25.6 ± 9.0%, *p* < 0.05). In addition, RA conduit function (passive EF, ɛe, SRe) was more impaired in HCM patients compared to HTN patients (*p* < 0.05). However, RA booster pump function (RA booster EF, ɛa, SRa) was preserved in both HCM and HTN patients (*p* > 0.05).

### 3.4. LA-LV coupling

**Figure 3:**
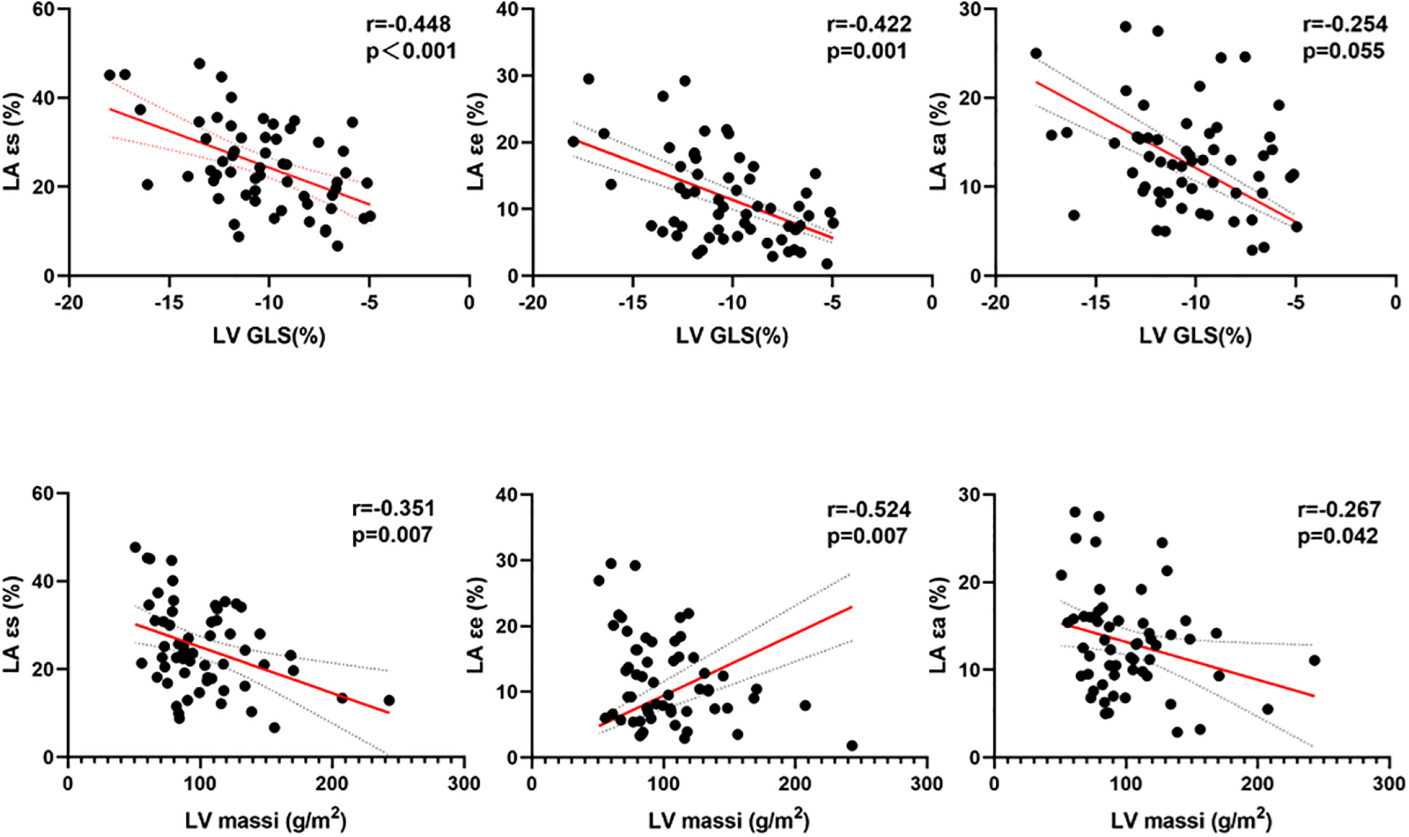
Correlations between LA strains and LV massi and LV GLS in patients with HCM. LA, left atrial; ɛs, total strain; ɛe, passive strain; ɛa, active strain; LV, left ventricular; massi, mass indexed by body surface area; GLS, global longitudinal strain.

Significant correlations were found between LA reservoir strain (ɛs), conduit strain (ɛe), and booster pump strain (ɛa) with LV EF, LV massi, LV MWT, global longitudinal strain parameters, and native T1 in HCM patients (*p* < 0.05; Table 4, Figure 3). The only correlations present in HTN were those between LA reservoir strain (ɛs) and booster pump strain (ɛa) with LV GLS (*p* < 0.05; Table 4).

**Table 4:** Correlations between LA strains and LV functional and deformation parameters in HCM and HTN patients.

	LA *ɛ*s, %		LA *ɛ*e, %		LA *ɛ*a, %	
	*r*	*p*	*r*	*p*	*r*	*p*
**HCM**						
LV EF, %	0.415	**0**.**001**	0.363	**0**.**005**	0.352	**0**.**007**
LV massi, g/m^2^	−0.351	**0**.**007**	−0.524	**0**.**007**	−0.267	**0**.**042**
LV EDD, mm	0.043	0.751	0.160	0.229	−0.033	0.805
LV MWT, mm	−0.483	**<0**.**001**	−0.399	**0**.**002**	−0.399	**0**.**009**
GLS,%	−0.448	**<0**.**001**	−0.422	**0**.**001**	−0.254	0.055
sGLSR, s^−1^	−0.487	**<0**.**001**	−0.352	**0**.**007**	−0.448	**0**.**004**
dGLSR, s^−1^	0.352	**0**.**007**	0.214	0.106	0.378	**0**.**003**
Native T1, ms	−0.325	**0**.**014**	−0.288	**0**.**031**	−0.265	**0**.**049**
**HTN**						
LV EF, %	0.110	0.477	−0.134	0.387	0.275	0.070
LV massi, g/m^2^	−0.078	0.615	−0.021	0.894	−0.132	0.393
LV EDD, mm	0.108	0.484	0.159	0.303	−0.037	0.810
LV MWT, mm	−0.178	0.247	−0.033	0.833	−0.256	0.094
GLS, %	−0.342	**0**.**023**	−0.067	0.666	−0.488	**0**.**001**
sGLSR, s^−1^	−0.077	0.619	−0.058	0.710	−0.051	0.742
dGLSR, s^−1^	−0.063	0.683	−0.214	0.164	0.172	0.264
Native T1, ms	−0.156	0.386	−0.103	0.570	−0.079	0.661

Bold values indicate statistical significance.

HCM, hypertrophic cardiomyopathy; HTN, hypertension; LV, left ventricular; EF, ejection fraction; massi, mass indexed by body surface area; EDD, end-diastolic diameter; MWT, maximal wall thickness; GLS, global longitudinal strain; sGLSR, peak systolic global longitudinal strain rate; dGLSR, peak diastolic global longitudinal strain rate; LA, left atrial; ɛs, total strain; ɛe, passive strain; ɛa, active strain.

### 3.5. Intra-observer and inter-observer reproducibility

**Figure 4:**
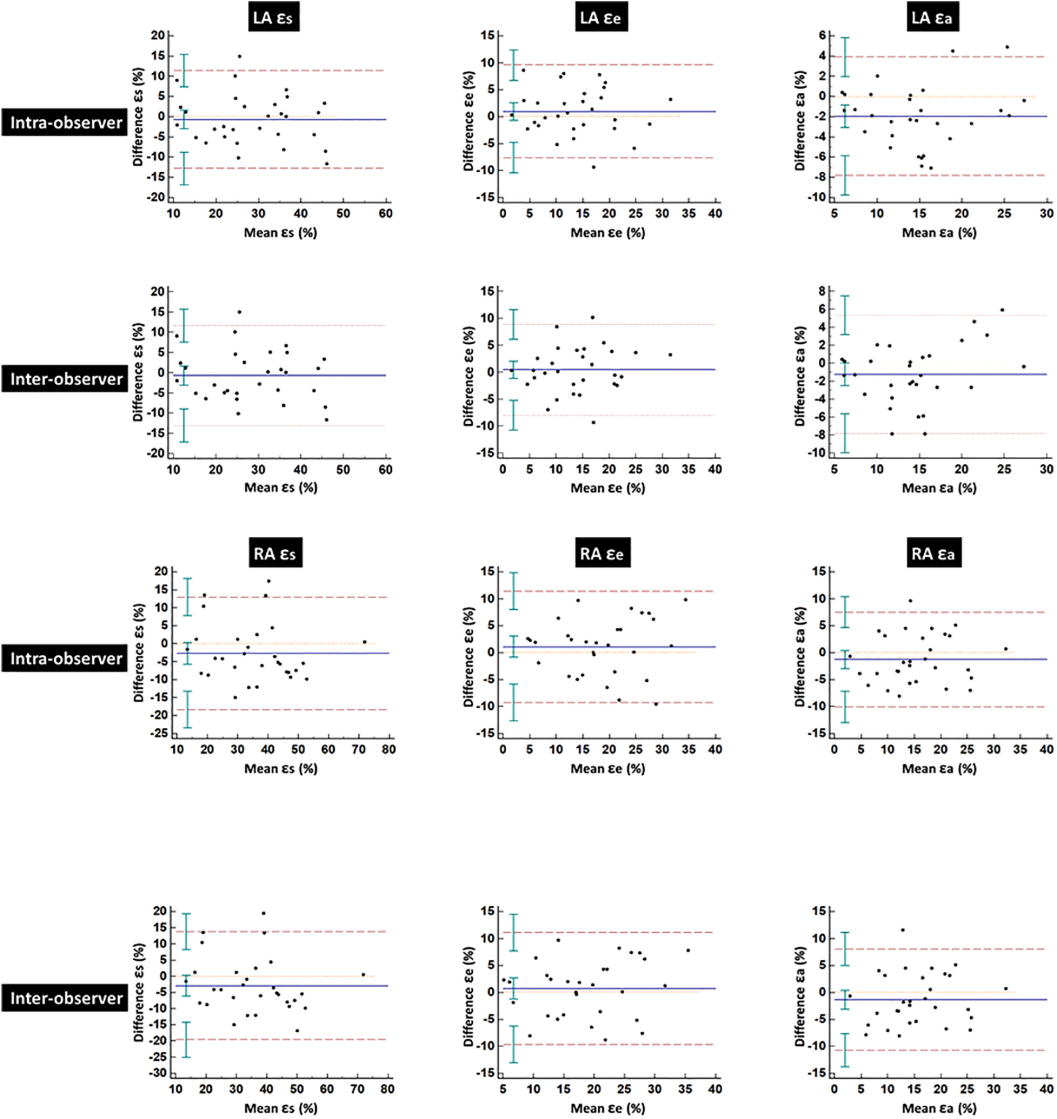
Bland–Altman plots for intra- and inter-observer variability obtained for global left and right atrial strains. LA, left atrial; RA, right atrial; ɛs, total strain; ɛe, passive strain; ɛa, active strain.

Table 5 summarizes the ICCs and CoVs of LA and RA global strain and SR parameters derived using CMR-FT. Bland–Altman plots for global strain measurements of LA and RA are shown in Figure 4. Both LA and RA strains and SR parameters demonstrated good intra- and inter-observer reproducibility values (intra ICC: 0.794–0.852, inter ICC: 0.752–0.845). Intra- or inter-observer analysis results for LA strains and SRs showed much higher reproducibility than RA strains and SRs. The lowest reproducibility was observed in RA SRe at the inter-observer level (ICC: 0.752).

**Table 5:** Reproducibility of global strain measurements performed using CMR-FT.

	Intra-observer reproducibility			Inter-observer reproducibility		
	ICC	(95% CI)	CoV (%)	ICC	(95% CI)	CoV (%)
LV GLS, %	0.937	0.873–0.970	10.81	0.910	0.818–0.956	12.09
RV GLS, %	0.898	0.797–0.950	11.56	0.891	0.784–0.947	15.28
LA ɛs, %	0.852	0.713–0.926	21.53	0.845	0.700–0.923	21.93
LA ɛe, %	0.827	0.671–0.913	29.79	0.820	0.657–0.910	27.90
LA ɛa, %	0.838	0.568–0.931	20.14	0.813	0.635–0.908	23.13
LA SRs, s^−1^	0.840	0.691–0.920	23.80	0.828	0.673–0.914	25.22
LA SRe, s^−1^	0.849	0.708–0.925	29.05	0.823	0.664–0.911	29.95
LA SRa, s^−1^	0.811	0.642–0.905	25.91	0.803	0.624–0.901	24.04
RA ɛs, %	0.819	0.651–0.910	22.22	0.800	0.617–0.900	23.61
RA ɛe, %	0.803	0.630–0.901	27.92	0.794	0.613–0.896	27.82
RA ɛa, %	0.793	0.611–0.895	28.31	0.761	0.559–0.878	29.21
RA SRs, s^−1^	0.797	0.617–0.898	24.25	0.766	0.566–0.881	25.13
RA SRe, s^−1^	0.767	0.506–0.890	29.26	0.752	0.357–0.896	30.25
RA SRa, s^−1^	0.794	0.615–0.896	28.29	0.765	0.567–0.880	29.31

LV, left ventricular; RV, right ventricular; GLS, global longitudinal strain; LA, left atrial; RA, right atrial; ɛs, total strain; ɛe, passive strain; ɛa, active strain; SRs, total strain rate; SRe, passive strain rate; SRa, active strain rate; ICC, intra-class correlation coefficient; CoV, coefficient of variation.

## 4. Discussion

The current study compared LA function and LA-LV coupling in patients with HCM and HTN who have preserved LV EF using CMR-FT and investigated the feasibility of CMR-FT in evaluating RA deformation. The main findings are as follows: (1) LA reservoir, conduit, and booster pump functions were impaired in both HCM and HTN patients, with reservoir and conduit functions more impaired in HCM patients; (2) HCM and HTN exhibited different LA-LV couplings, with significant correlations between LA strains (ɛs, ɛe, ɛa) and LV compliance and systolic strain parameters in HCM, while in HTN, correlations were only found between LA reservoir strain (ɛs) and booster pump strain (ɛa) with LV GLS; (3) RA reservoir and conduit strains were reduced prior to RV dysfunction in both HCM and HTN patients, while booster pump function was preserved.

Based on the structural parameter data, LA morphological remodeling was observed in our patients, as evidenced by enlarged LA size and volume. LA reservoir and conduit functions were impaired in both HCM and HTN patients, which was consistent with previous studies (21, 30, 31). The potential mechanisms are associated with increased LV wall stiffness, elevated LV filling pressure, and impaired LA-LV coupling (11, 32). Furthermore, we discovered that LA reservoir and conduit functions were more severely impaired in HCM patients compared to those with HTN, which may have been due to greater LV wall thickening and more severe diastolic dysfunction in HCM. In addition to LV diastolic dysfunction, studies have shown that LA dysfunction is also correlated with LV fibrosis (33). Contractile function, which is primarily modulated by intrinsic atrial contractility and related to LA size, has been reported to be inconsistent, with some studies reporting normal (31, 34, 35), increased (15, 36), or reduced (37) contractile function. This inconsistency may be attributed to different inclusion criteria. Impaired booster pump function was observed in both HCM and HTN patients in our study, reflecting a state of “decompensation” and a progressive stage of LA dysfunction in the study population (3). Additionally, a trend of more severe contractile function impairment was noted in HCM patients compared to those with HTN, although the difference was not statistically significant.

Interestingly, the present study showed that different atrio-ventricular interactions occurred in the two different diseases. Significant correlations were found between LA strains (ɛs, ɛe and ɛa) and LV compliance (LV massi, LV MWT) as well as systolic parameters (LV EF, GLS, sGLSR and native T1) in HCM patients. In contrast, fewer correlations were found in HTN, with the only correlation observed between LA strains (ɛs, ɛa) and impaired LV GLS. These findings indicate that HCM and HTN display different patterns of LA-LV coupling, which is in line with previous STE-based studies (8, 34). In a similar CMR study, Zhou et al. also reported that LA function correlated with the severity of LV diastolic function in HCM, but more closely with LV systolic function in HTN (31). However, another CMR study conducted by Song et al. (38) demonstrated that LV diastolic deformation indices were significantly correlated with LA reservoir and conduit function in HTN patients. The discrepancy may be ascribed to differences in the patient population stages. As previously mentioned, the impaired booster pump function indicates a progressive stage in the present study. Thus, we speculate that normal LA-LV interaction may be disrupted during a progressive stage of hypertension. Interestingly, although normal LA-LV interactions were not discovered, booster pump strain (ɛa) was demonstrated to be associated with impaired LV GLS. This finding suggests that LA contractile function might be a superior index reflecting atrio-ventricular state in the advanced stages of hypertension. In this context, we propose that future studies should pay more attention to abnormal LA-LV coupling, which may enhance our understanding of the course of hypertension.

RV hypertrophy and dysfunction are frequently observed in both HCM and systemic hypertension (39, 40). Previous studies have reported that RV global strain is also deteriorated, possibly due to ventricular interaction (2, 19). The present study data showed that HCM patients had impaired RV GLS with preserved RV EF, while RV GLS in HTN did not significantly differ from healthy controls. To the best of our knowledge, there were fewer related studies reporting on RA strain determined using CMR-FT in HCM and HTN. In the present study, RA enlargement and decreases in reservoir and conduit strains were observed in both HCM and HTN patients, demonstrating that RA structural remodeling and dysfunction can occur before RV dysfunction in the disease process. This finding is consistent with previous STE studies, which have shown that RA reservoir and conduit functions were impaired in HCM patients and that longitudinal strain was damaged in untreated and uncontrolled hypertensive patients (19, 41). The potential mechanism could involve a constant increase in RV filling pressure, similar to that in the LA and LV. Preserved RA contractile function (ɛa) and increased late diastolic SRa observed in our study patients represent a compensatory reaction to maintain stroke volume and RV filling with mild diastolic dysfunction (31).

There are some limitations to our study. First, the study sample size was relatively small, and the cohort predominantly consisted of Chinese participants. Therefore, these findings require further validation and confirmation in larger-scale studies with a more diverse population. Second, subgroup analysis was not performed in this study due to its small sample size. The HCM cohort included patients with and without obstruction of the LV outflow tract, and patients with and without LV hypertrophy were included in the HTN cohort. Third, since some of our patients did not undergo the hematocrit (HCT) laboratory test on the day of the CMR examination, extracellular volume fraction (ECV) data could not be obtained. Lastly, due to the thin RA wall and the tricuspid valve attachment point not being as clearly visible as the bicuspid valve, tracking the RA endocardial and epicardial borders was more challenging than tracking those of the LA. Furthermore, while the LA outline was tracked on two long-axis views, the RA outline was tracked on only one long-axis view. Consequently, RA strains and SRs demonstrated weaker intra- and inter-observer reproducibility compared to the LA in the present study.

## 5. Conclusion

This study demonstrated the feasibility of using CMR-FT in evaluating RA deformation and provided insight into the differences in LA function and LA-LV coupling between patients with HCM and HTN. LA reservoir, conduit, and booster pump functions were impaired in both HCM and HTN patients, with reservoir and conduit functions more impaired in HCM patients. HCM and HTN exhibited different patterns of LA-LV coupling, which may have clinical implications for understanding the disease course and developing targeted therapies. Furthermore, RA structural remodeling and dysfunction were found to occur before RV dysfunction in both HCM and HTN patients. Future larger-scale studies with diverse populations are needed to validate and confirm these findings, potentially leading to improved understanding and management of these diseases.

## Data Availability

The raw data supporting the conclusions of this article will be made available by the authors, without undue reservation.
